# Acupuncture therapy strategy options in postoperative management after laparoscopic cholecystectomy: A protocol for systematic review and Bayesian meta-analysis

**DOI:** 10.1097/MD.0000000000024199

**Published:** 2021-01-08

**Authors:** Dong-qin Zhao, Guang-yu Qian, Jing Jin, Yin-ping Yao, Xing-mao Bian, Wei-ping Zhang

**Affiliations:** a Zhuji People's Hospital of Zhejiang Province; b Zhuji Affiliated Hospital of Shaoxing University, Zhuji, 311800, China.

**Keywords:** acupuncture therapy, analgesia, enhanced recovery after surgery, laparoscopic cholecystectomy, network meta-analysis, postoperative nausea and vomiting

## Abstract

**Background:**

Laparoscopic cholecystectomy (LC) is a common surgery accompanied by some unpleasant adverse effects. Clinical trials indicated that acupuncture therapy may help reduce complications in LC. However, no systematic reviews have been conducted on the topic. Therefore, we will evaluate the current evidence and provide a rank for the efficacy of acupuncture therapy in LC by performing Bayesian network meta-analysis.

**Methods:**

A total of 9 databases will be searched from inception to 10 December 2020. Randomized control trails met the criterion will be included. Quality evaluation of included studies will be performed using Cochrane risk-of-bias tool. STATA 14.0, Addis 1.16.8, R 3.6.3, and OpenBUGS 3.2.3 will be used to conduct pairwise meta-analysis and network meta-analysis. The evidence will be assessed by the Grades of Recommendations Assessment Development and Evaluation.

**Results:**

This review will be based on clinical evidence to choose the best choice of acupuncture treatment for LC. And the results will be submitted to a peer-reviewed journal for publication.

**Conclusion:**

Through this systematic review, we will summarize the best available evidence of acupuncture therapy in LC and help to improve the clinical decision-making ability in LC domain.

**Systematic review registration:**

The protocol has been registered on INPLASY2020120056.

## 1 Introduction

Nowadays, laparoscopic cholecystectomy (LC) has been the gold standard of in treating patients with symptomatic cholelithiasis and wildly used around world.^[1]^ However, the adverse effect of LC still cannot be ignored, because it will delay discharge, increase medical cost, and decline the satisfactory of patients.^[2]^ Among all adverse effects, postoperative pain (POP) and postoperative nausea and vomiting (PONV) are the most common complications with the incidence of 50% to 70% and 45% to 75%, respectively.^[3,4]^ Non-steroidal anti-inflammatory drugs and opioid have been used to relieve POP and a series of antiemetics have been used to prevent PONV.^[5,6]^ Nevertheless, POP may still occur and afflict patient in postoperative 1 month after LC, with the cost-effectiveness less than satisfactory.^[7]^ In particularly, only one-third of patients can benefit from prophylactic antiemetics and PONV within 24 hours still occurs in 25% to 30% of patients.^[8,9]^ In addition, other adverse effects like respiratory depression, gastrointestinal ulceration, headache, and malignant ventricular arrhythmias, have triggered growing concern.^[10,11]^ Therefore, finding novel and effective therapies to enhance recovery after LC has become an integral part of postoperative rehabilitation.

Some clinicians shift their attentions to complementary and alternative therapy, and find that acupuncture therapy is a potential treatment. Meta-analyses show that acupuncture could relieve POP and PONV in craniotomy, total knee arthroplasty, and tonsillectomy.^[12–14]^ And similar results have been proved in LC.^[15–17]^ However, it has not been summarized into a systematic review about the efficacy of acupuncture therapy in LC. And we still do not know whether all kinds of clinical acupuncture treatments can account for postoperative management after LC. Meanwhile, there is still controversial whether non-invasive regimens are as effective as invasive ones.^[18]^ To handle them with proper assessments will help remove the fetters of acupuncture application in enhanced recovery after surgery (ERAS).

In this systematic review, we will deal with 3 issues based on the above situation. First, which acupuncture treatments may help ERAS after LC; second, can non-invasive regimens comparable with invasive ones; third, patients can get benefits from which acupuncture treatment most. For the sake of solving these issues, we will use network meta-analysis (NMA) based on Bayesian model and hope this study could inspire relevant study.

## 2 Methods

The protocol has been registered on INPLASY (https://inplasy.com/) with the identification number INPLASY2020120056. Ethical approval is unnecessary, because this is a systematic literature research. We will follow the statement of Preferred Reporting Items for Systematic review and Meta-Analysis Protocols (PRISMA-P).^[19]^

### 2.1 Eligibility criteria

#### 2.1.1 Type of study

Peer-reviewed randomized control trails written in English or Chinese will be included.

#### 2.1.2 Participants

Patients undergoing LC will be included regardless of age, gender, race, and diseases.

#### 2.1.3 Interventions

Patients in experimental group used any acupoint stimulation regimens will be included, for instance, manual acupuncture, electroacupuncture, moxibustion, transcutaneous electric nerve stimulation, and acupressure. Considering that clinicians may combine acupuncture with medications, those studies will also be included. Patients in control group used usual care, placebo/sham acupuncture, medications. But other complementary or alternative therapies were excluded (e.g., herbal therapy, mindfulness).

#### 2.1.4 Outcomes

The primary outcomes are pain scores and incidence of PONV within 24 hours. Secondary outcomes include consumption of antiemetics and analgesics, and safety of acupuncture regimens. Other outcomes will also be assessed if necessary.

### 2.2 Search strategies

A comprehensive search of 9 electronic databases will be performed including PubMed, Cochrane library, Web of Science, Ebsco, Embase, China National Knowledge Infrastructure (CNKI), Wanfang Database, VIP Database, and China Biology Medicine disc (CBM) from setup time to 30 November 2020. The search strategy will contain both LC and acupuncture treatments including “cholecystectomy, laparoscopic,” “laparoscopic cholecystectomy,” “acupuncture,” “electroacupuncture,” “acupuncture therapy,” and similar terms. MeSH terms were used and combined with free-text words. Search strategy will be adjusted depending on each database. Table 1 has given a detailed search strategy of PubMed.

**Table 1:** Search strategy of PubMed.

Search number	Query
#1	acupuncture [MeSH Terms]
#2	moxibustion [MeSH Terms]
#3	electroacupuncture [MeSH Terms]
#4	acupuncture therapy [MeSH Terms]
#5	acupressure [MeSH Terms]
#6	transcutaneous electric nerve stimulation [MeSH Terms]
#7	acupuncture [Title/Abstract]
#8	moxibustion [Title/Abstract]
#9	electroacupuncture [Title/Abstract]
#10	acupressure [Title/Abstract]
#11	transcutaneous electric nerve stimulation [Title/Abstract]
#12	transcutaneous electrical acupoint stimulation [Title/Abstract]
#13	#1 OR #2 OR #3 OR #4 OR #5 OR #6 OR #7 OR #8 OR #9 OR #10 OR #11 OR #12
#14	laparoscopic cholecystectomy [MeSH Terms]
#15	laparoscopic cholecystectomy [Title/Abstract]
#16	cholecystectomies, laparoscopic [Title/Abstract]
#17	laparoscopic cholecystectomies [Title/Abstract]
#18	celioscopic cholecystectomies [Title/Abstract]
#19	celioscopic cholecystectomy [Title/Abstract]
#20	#14 OR #15 OR #16 OR #17 OR #18 or #19
#21	#13 AND #20

### 2.3 Study selection

**Figure 1:**
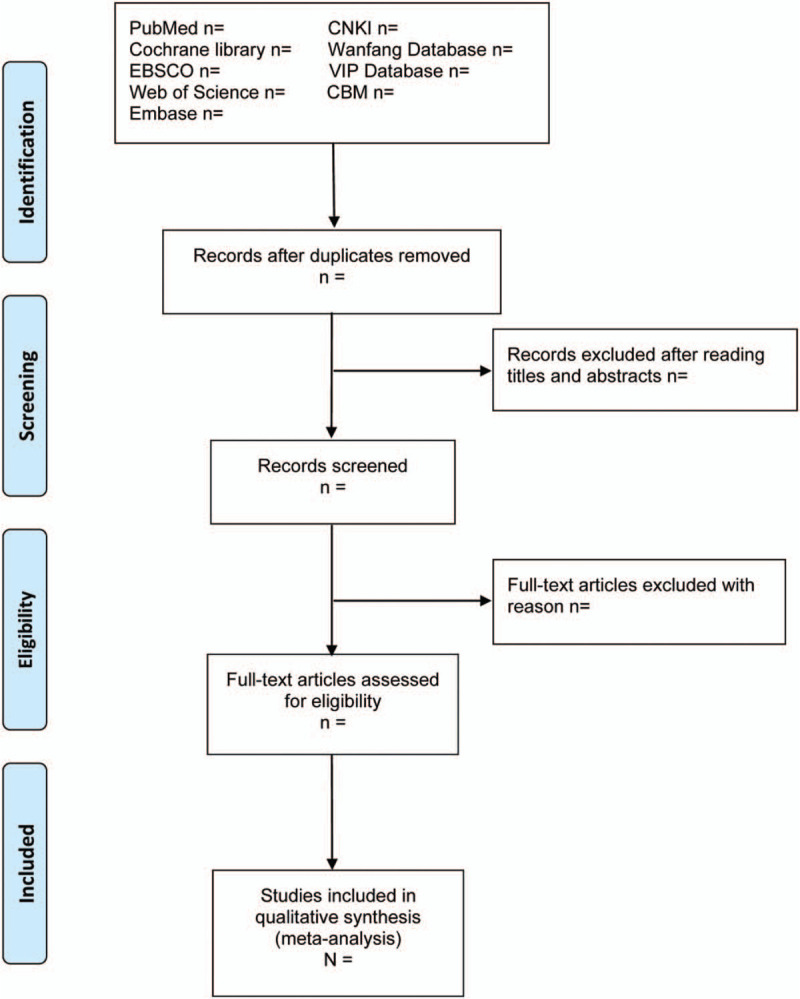
PRISMA flow diagram of the study selection process.

Two reviewers will scan all studies independently and a third reviewer will request adjudications if necessary. Only the most informative and complete study of any duplicate publications will be selected. The process of screening will be shown by PRISMA flow chart as Figure 1.

### 2.4 Data extraction

Two reviewers will extract the following data into a database created respectively:

(1) title, first author, publication year, registration of clinical trial registry;
(2) sample size, gender, diseases, race, postoperative analgesia, American Society of Anesthesiologists Class, types of intervention and acupoints;
(3) the incidences of PONV and score of POP, the assumption of antiemetics and analgetic.

In addition, GetData Graph Digitizer will be used to extract information from figure data. The third reviewer is the referee in case of disagreements.

### 2.5 Risk of bias assessment

Cochrane risk-of-bias tool (ROB 2.0) will be used to evaluate the quality.^[20]^ There are 5 domains including randomization process bias, deviations bias from intended interventions, missing outcome data bias, measurement bias of the outcome, selection bias of the reported result. Finally, an overall risk of bias will be given based on above bias. Two reviewers will use ROB 2.0 to assess all matched studies and the third reviewer will request adjudications if necessary.

### 2.6 Statistical analysis

#### 2.6.1 Pairwise meta-analysis

Only 3 or more studies reported the same outcome will be performed by Stata 14.0. Odds ratio (OR) and 95% confidence interval (CI) and standard mean difference (SMD) with the 95% CI will be adopted to calculate dichotomous variables and continuous variables, respectively. *I*-square will be used to statistic heterogeneity. It is regarded as the boundary that if *I*-square ≤ 50%, a fixed will be more suitable, otherwise a random effect model will be performed. And sensitivity analysis will be accomplished if sufficient studies are available before selecting model. When pairwise comparison studies ≥ 10, a Begg's testing will be performed to explore the publication bias. Subgroup analysis and regression analysis will be conducted according to elements like acupuncture regimens and publication year if reasonable.

#### 2.6.2 NMA

NMA will be performed by Addis1.16.8, OpenBUGS 3.2.3, R3.6.3, and STATA 14.0. Our reasonable PICOS will help lower the clinical heterogeneity by limiting the participants’ characteristics, interventions, and outcomes of the included trials. R will used to assess the methodological heterogeneity. Convergence will be conducted by using the Brooks–Gelman–Rubin method. By calculating the Potential Scale Reduction Factor (PSRF), the convergence can be obtained. And PSRF < 1.05 indicates acceptable convergence. Node spilt analysis through the comparison of direct and indirect effect will be performed to assess inconsistency. League figures will be used to demonstrate the results of multiple treatment comparisons. The surface under the cumulative ranking curve (SUCRA) values will point out the best choice based on the included studies.

### 2.7 Quality of evidence

Grades of Recommendations Assessment Development and Evaluation (GRADE) guidelines will be used to evaluate the quality of evidence through 5 factors to lower it and the quality will be graded in 4 levels.

## 3 Discussion

Study highlighted that 96% cholecystectomy were performed by laparoscopy with the 70% diagnosis were biliary colic or acute cholecystitis.^[21]^ Thus, the importance of perioperative management of LC is obvious. Since ERAS was proposed, clinicians have achieved consensus that it could reduce postoperative complications, accelerate recovery as well as improve perioperative outcomes.^[22,23]^ In Chinese Medicine theory, acupuncture assists in body recovery through a kind of holistic modulation translating to body balance. Now it is wildly applied in perioperative period and clinicians would try to use the natural therapy for ERAS.^[24]^ However the best practice of acupuncture treatments in patients with LC has not been clarified. Clinicians are in urgent need of high-quality evidence of acupuncture in LC and they need the assessment of therapeutic value of different acupuncture regimens. Therefore, we will make a NMA to solve the pain point as well as provide a credible rank of various acupuncture therapies. There are several limitations that we anticipate. The parameter of stimulation (e.g., time and intensity) may influence the effect of acupuncture therapy, subgroups will be set to explore the homogeneity as necessary. Besides, we only will include studies written in English and Chinese, it may lead to some bias. But by publishing this meta-analysis, we will attract more like-interest researchers to participate the program to reduce the bias.

The results will be published in relevant journal and it will find out which acupuncture interventions have the best efficacy and safety in LC recovery. We will update the protocol if new studies appear in the future.

## Author contributions

**Data curation:** Dong-qin Zhao, Wei-ping Zhang.

**Formal analysis:** Dong-qin Zhao, Guang-yu Qian, Jing Jin.

**Funding acquisition:** Guang-yu Qian.

**Investigation:** Jing Jin, Yin-ping Yao, Guang-yu Qian.

**Methodology:** Dong-qin Zhao, Xing-mao Bian, Wei-Ping Zhang.

**Project administration:** Wei-ping Zhang.

**Resources:** Dong-qin Zhao, Xing-mao Bian.

**Software:** Dong-qin Zhao, Wei-ping Zhang.

**Supervision:** Yin-ping Yao, Xing-mao Bian.

**Writing – original draft:** Dong-qin Zhao, Guang-yu Qian.

**Writing – review & editing:** Dong-qin Zhao, Wei-ping Zhang.
